# Navigating the complexity: a comment on difficulties in conducting systematic reviews to evaluate the effectiveness of behaviour change interventions

**DOI:** 10.1186/s12966-025-01857-x

**Published:** 2025-12-17

**Authors:** Sarah Rhodes, Ukachukwu Abaraogu

**Affiliations:** 1 Centre for Biostatistics, Division of Division of Population Health, Health Services Research & Primary Care, School of Health Sciences, University of Manchester, Oxford Road, Manchester, M13 9PL UK; 2 Division of Biological Sciences and Health, School of Health and Life Sciences, University of the West of Scotland, Stephenson Place, Hamilton International Technology Park, Technology Avenue, South Lanarkshire, G72 0LH UK; 3 Department of Medical Rehabilitation, Faculty of Health Sciences, College of Medicine, University of Nigeria, 1 College Road, New Layout, Enugu, 401101 Nigeria

**Keywords:** Health behaviour change, Systematic review, The behaviour change technique taxonomy

## Abstract

Behaviour change interventions are widely applied across diverse domains to modify behaviours and improve outcomes, including but not limited to physical activity, sedentary behaviour, and nutrition. Systematic review of trials of behaviour change interventions often aim to determine both their overall effectiveness and the specific components driving behaviour change. The Behaviour Change Technique Taxonomy v1 (BCTTv1) is a useful framework for coding the individual Behaviour Change Techniques (BCTs) within an intervention. This commentary highlights key methodological challenges in conducting a systematic review of behaviour change interventions. It calls for collaboration in developing clear and consistent methodological guidance to enhance the rigor of systematic reviews to optimise behaviour change interventions.

## Introduction

Behaviour change interventions are commonly used to promote health-enhancing behaviours, support chronic disease management, prevent illness, improve mental well-being, and foster sustainable lifestyle practices, including addressing physical activity, sedentary behaviour, nutrition, and related health outcomes. Many systematic reviews look to combine data from randomised controlled trials to work out firstly whether the behaviour change interventions are effective and if so, which elements of these interventions contribute to that effect.

The Behaviour Change Technique Taxonomy v1 (BCTTv1) [1] is a groundbreaking structured framework for identifying, classifying and describing the active components driving behaviour change interventions. It provides a common language to understanding Behaviour Change Techniques (BCTs), allowing the research community to code the BCTs within behaviour change interventions in a consistent way. For systematic reviewers, this framework should be a game changer, offering a structured way to conduct analysis, with the ultimate goal of designing the “optimal intervention.” The promise of an optimal intervention, using only those elements that are effective and avoiding those that aren’t, is the holy grail, sold to excited funders, patients and other stakeholders. Optimistic systematic reviewers regularly embark on the mammoth task of searching for trials and coding every behaviour change component.

## Methodological challenges

We have been those optimistic reviewers. We conducted a systematic review of behaviour change interventions to increase habitual physical activity in patients with Intermittent Claudication [2]. We completed the mammoth task, and would do the same again, but would like to report that it was very messy and we didn’t quite find the holy grail. We aren’t alone in this. A scoping review performed in 2018 [3] looked at methods to identify and evaluate effective BCTs for specific behaviours and contexts and concluded that only weak conclusions could be drawn regarding the effectiveness of specific BCTs and BCT combinations in the majority of cases.

The use of BCT codes in a systematic review brings some specific challenges. Firstly, there is the expectation that coding individual intervention components will lead to information about which components are driving intervention effectiveness and which ones are redundant and can be removed.

In an attempt to do this, some large systematic reviews use complex components based methods [4] that use regression models to estimate the individual effect of each BCT or machine learning methods [5] that develop algorithms to predict the effectiveness of a combination of BCTs in a given context. These methods are most likely to work well when the number of studies is large, the number of candidate BCTs is small and methodology is similar amongst trials. However, more commonly, reviews are small and the BCTs used are varied, meaning that authors struggle to make useful conclusions about which components are most effective. Further, interventions are typically poorly reported and don’t include detail on frequency of use or fidelity to their protocol which introduces noise and limits the reliability of conclusions.

In our case, what we ended up with was a set of 15 trial results looking at habitual physical activity in the short term, our primary analysis, and a total of 46 individual BCTs used in at least one intervention. Effects were remarkably consistent across our 15 results, and it was clear that on average these interventions were beneficial. However, when we used meta-regression and network meta-analysis with the aim to estimate the average effect of including individual BCTs or sets of BCTs, none stood out from any other as being associated with larger than average effect. It should also be noted that comparing interventions with and without certain BCTs in this way alone does not allow causal conclusions and can only suggest associations to be investigated further; perhaps another over-optimistic expectation for some systematic reviewers. While the BCT coding will of course help others to identify elements that are commonly used in this type of intervention, which will be useful to build their own, the evidence for the effectiveness of each individual BCT component is still lacking and one may question the work/benefit ratio of coding every BCT component in such meticulous detail.

In addition to the challenge of identifying the most effective ingredients, there were further complexities that we believe are specific to reviews of behaviour change interventions. One such complexity is the ‘control’ arm. In our review, we included any comparison between a behaviour change intervention and any control; taking a control to be an intervention that contained fewer or no BCTs. Frequently some individual BCTs appeared in both intervention and control of the same trial, possibly in differing formats or doses. Our approach to this was to subtract the control arm BCTs from the intervention BCTs and focus only on the remaining BCTs unique to the intervention arm. However, we are aware that this approach is not ideal because it assumes that the component effect of each BCT is identical in both intervention and control arms. This is unlikely to be the case. Individual BCTs may interact with each other, meaning that they may be more or less effective when combined with different sets of BCTs. Furthermore, this over-simplification ignores the difference in format and dose in which the BCTs were used in the intervention and control arms which may change the effectiveness of the BCT.

The inextricable link between intervention and outcome is another aspect of our review that sent us round in circles. A behaviour change intervention is designed to target a specific behaviour. Our initial strategy was to exclude studies where our target behaviour, habitual physical activity, was not reported on. However, our patient and public contributors told us that the most important outcomes to them are quality of life domains such as function and pain; and we became aware that there were studies that target increased physical activity (without reporting on it as an outcome) and had important data on the Patient Reported Outcomes (PROs). Furthermore, excluding studies on the basis of outcome would have put our review at risk from outcome reporting bias. After much debate, we ended up modifying our inclusion criteria, getting extra time from our funder, and revisiting our excluded studies.

## Looking for solutions

Systematic reviews of behaviour change interventions are complex, presenting unique challenges that reviewers and researchers tackle with conflicting approaches, often in isolation. The ‘effectiveness ratio’ [6] has been used by some authors, defined as the proportion of interventions with a statistically significant result, out of all interventions where the BCT was used. The ‘promise ratio’ [7] is an alternative metric that compares the number of times a BCT appears in a ‘very or quite promising intervention’ divided by the number of times it appears in a non-promising intervention, with a measure of promise defined by comparing intervention group improvements to control group improvements. A ‘structured narrative moderator analysis’ [8] plots studies in order of effect size alongside a mapping of component BCTs; this allows reviewers to discuss narratively the BCTs and combinations of BCTs most frequently appearing in interventions associated with the large effect sizes. These novel approaches each have merits and are appealing due to their simplicity, but they have limitations such as ignoring study precision.

In addition to soloed and inconsistent approaches, we have observed that systematic reviewers often do not report important methodologies used and decisions made in extracting individual BCTs in different trial arms. It is also often unclear how BCT numbers were finalised between active and comparison arms of trials in systematic reviews of behaviour change interventions. This makes it impossible to infer transparency and reproducibility of the process.

## The future

The recent introduction of new BCT ontology [9] provides additional BCTs and promises more structured BCT groupings and improved ability to code via a computer, alongside links between BCT use and theoretical mechanisms [10]. Furthermore, rapidly advancing Artificial Intelligence (AI) methods are likely to change the way that reviews are conducted and analysed [11]. With these improvements in place, the research community must work together without delay to realise their full value in systematic reviews.

Personally, we would really like to see more specific guidance for systematic reviewers of behaviour change interventions that helps them to navigate the complexities highlighted here. We already have plans to start this process. We hope that by reviewing current practice we can evidence the extent to which problems exist and uncover novel ideas that may help to solve them. Through discussions with all relevant stakeholders including public, researchers and practitioners we hope to find out which issues to focus on and share ideas. Ultimately, we’d like to see a consensus process such as a Delphi survey to build recommendations that are recognised as useful by all stakeholders.

Any recommendations produced should be designed to help researchers to tailor their methods to the research question they want to answer, and the volume of evidence they expect to find. This will help reduce unnecessary time and resources spent extracting detail that isn’t required or planning analyses that are not feasible.

It is hoped that recommendations will help researchers move beyond over idealistic expectations about isolating the effectiveness of individual components and instead utilise all the available quantitative data alongside other information such as context and theoretical mechanism to produce useful evidence that will influence practice.

Such collective action would foster more efficient, rigorous, and unbiased methodologies, ultimately enhancing the quality of evidence synthesis. By addressing these challenges together, we can reduce research waste, ensure the reproducibility of findings and accelerate the development of effective behaviour change interventions.

## Conclusions

We would urge the community of behaviour change intervention researchers to collaborate on guidance on how to complete a methodologically rigorous systematic reviews of behaviour change interventions and synthesize evidence with precision, while harnessing the potential of AI methods and the new BCT ontology.

## Data Availability

Not applicable.

## Abbreviations

BCT: Behaviour change technique
BCTTv1: The Behaviour Change Technique Taxonomy v1
PROs: Patient Reported Outcomes
AI: Artificial Intelligence
